# Supplementary material for: A Global Screen for Assembly State Changes of the Mitotic Proteome by SEC-SWATH-MS
Source: Cell Syst. 2020 Feb 26;10(2):133–155.e6. doi: 10.1016/j.cels.2020.01.001 (PMC7042714; doi:10.1016/j.cels.2020.01.001)

Q9Y5S9 | RBM8A_HUMAN | RBM8A RBM8 HSPC114 MDS014
Monomer MW [kDa]: 19.889 Monomer expected elution fraction: 54

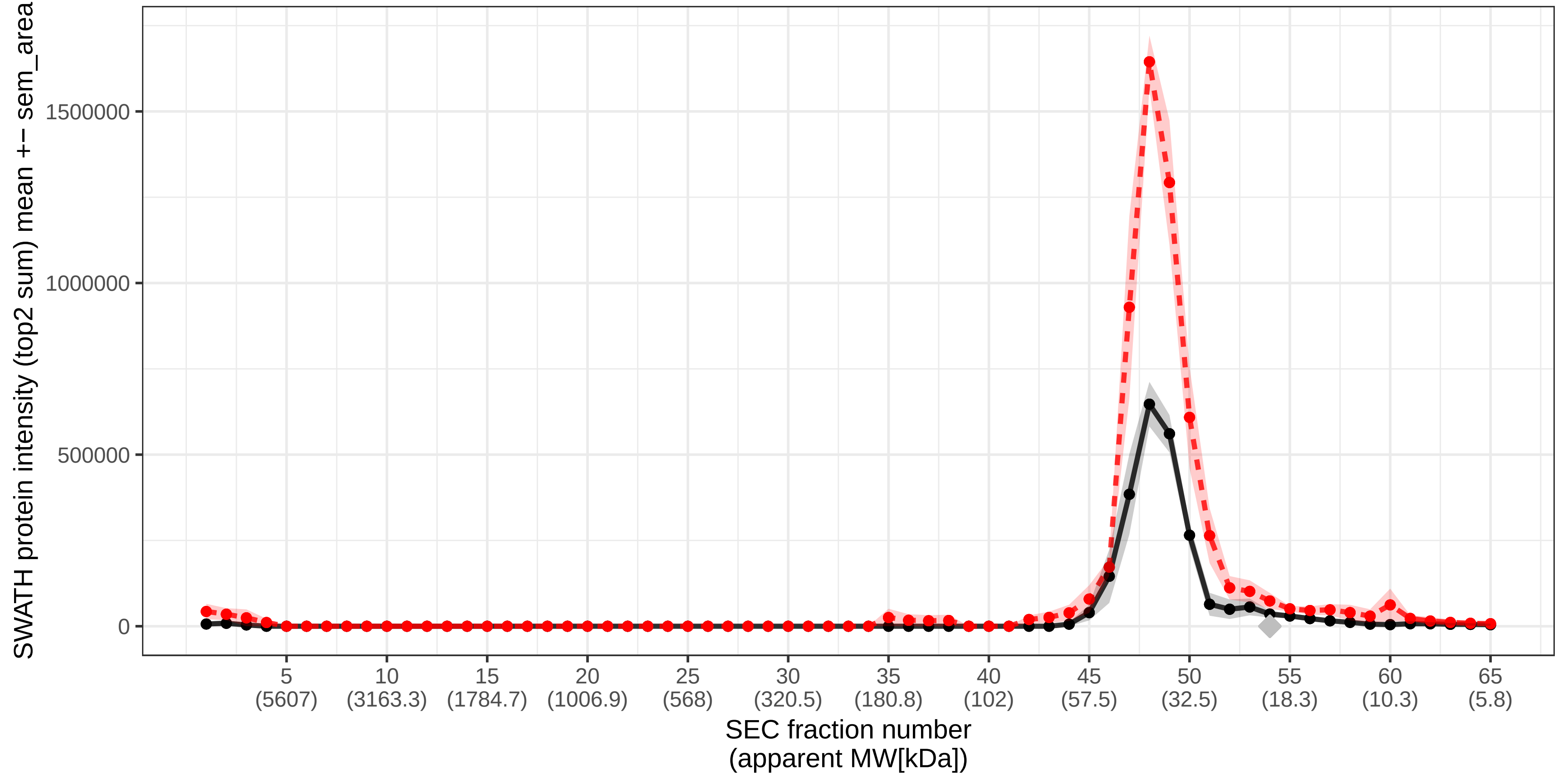

Supplement: Data S1. SEC-SWATH-MS Protein Chromatograms, Related to Figure 1 [file mmc6.zip › SECchrom_Q9Y5S9_RBM8A_HUMAN_RBM8A_RBM8_HSPC114_MDS014.pdf]
